# Knockdown of E2f1 by RNA interference impairs proliferation of rat cells *in vitro*

**DOI:** 10.1590/S1415-47572009005000104

**Published:** 2010-03-01

**Authors:** Luciana dos Reis Vasques, Regiane Simoni Pujiz, Bryan Eric Strauss, José Eduardo Krieger

**Affiliations:** Instituto do Coração, Escola de Medicina, Universidade de São Paulo, São Paulo, SPBrazil

**Keywords:** RNAi, shRNA, E2F1, proliferation, cancer

## Abstract

E2F1 plays a key role in cell-cycle regulation in mammals, since its transcription factor activity controls genes required for DNA synthesis and apoptosis. E2F1 deregulation is a common feature among different tumor types and can be a major cause of cell proliferation. Thus, blocking *E2F1* expression by RNA interference represents a promising therapeutic approach. In this study, the introduction of specific short hairpin RNAs (shRNAs) reduced *E2f1* expression by up to 77%, and impaired rat glioma cell proliferation by approximately 70%, as compared to control cells. Furthermore, we investigated the expression of *E2f1* target genes, *Cyclin A* and *Cyclin E*. *Cyclin A* was found to be down-regulated, whereas *Cyclin E* had similar expression to control cells, indicating that gene(s) other than *E2f1* control its transcription. Other E2f family members, *E2f2* and *E2f3*, which have been classified in the same subgroup of transcriptional activators, were also analyzed. Expression of both *E2f2* and *E2f3* was similar to control cells, showing no cross-inactivation or up-regulation to compensate for the absence of E2f1. Nevertheless, their expression was insufficient to maintain the initial proliferation potential. Taken together, our results suggest that shE2f1 is a promising therapy to control tumor cell proliferation.

E2F comprises a family of transcription factor proteins, with a pivotal role in controlling genes related to cell-cycle progression (Helin *et al.*, 1992; Kaelin Jr *et al.*, 1992; Shan *et al.*, 1992). Eight E2F family members have been identified so far, E2F1 to E2F8, whereas E2F1 to E2F6 share the same structure: conserved DNA binding and dimerization domains, and, except for E2F6, have domains for transactivation and binding Pocket Proteins (PP): p107, p130 and Rb (Retinoblastoma) (reviewed in Tsantoulis and Gorgoulis, 2005; DeGregori and Johnson, 2006). In general terms, the E2F family can be functionally classified in two subgroups, namely transcriptional activators (E2F1 to E2F3a) and repressors (E2F3b to E2F8). The E2F dimerization domain binds to members of the DP protein family, and the resulting complexes regulate overlapping gene collections (DeGregori and Johnson, 2006).

Despite Rb having been found to be associated with many members of the family, E2F1 is its main target (Wells *et al.*, 2003; Frolov and Dyson, 2004). Rb phosphorylation by Cyclin D/CDK4 and Cyclin E/CDK2, in late G1 phase, releases E2F transcription factors, thereby promoting expression of genes related to DNA synthesis and cell-cycle progression, resulting in cell proliferation (Polyak *et al.*, 1994; DeGregori *et al.*, 1995). The dissociation of E2F from pRb protein seems to be the main determinant in regulating cell proliferation, by permitting transactivation of genes such as *cyclin A*, *cyclin E*, *c-myb*, *cdc2*, *PCNA* and *thymidine kinase*, and committing cells to S phase (DeGregori, 2002).

The best characterized gene of the E2F family is E2F1, which plays a paradoxical role by acting in two opposing pathways: induction of cell cycle progression and apoptosis (Pierce *et al.*, 1999). E2F1, in response to DNA damage, can induce apoptosis by regulating related genes in a p53-dependent and p53-independent manner (Bates *et al.*, 1998; Irwin *et al.*, 2000; Lissy *et al.*, 2000; Moroni *et al.*, 2001).

Dysfunction of the intricate cell-cycle regulation pathways described above can exacerbate cell growth and, eventually, lead to cancer-cell development. In fact, deregulation of *E2F1* gene expression is a common event in the majority of tumors, where it appears over-expressed rather than mutated (Sherr, 1996; Dyson, 1998). E2F1 over-expression is due to a positive feedback loop created between this protein and its own promoter and due to high pRb phosphorylation levels or lack of functional pRb, both resulting in the liberation of E2F1. The main reasons for this hyperphosphorylation are high CDK4/6 and CDK2 activity, the absence of Cdk inhibitors (CDIs) and over-expression of Cyclins (reviewed in Halaban, 2005).

Since Rb is the most important PP and is preferentially associated with E2F1, inactivation of *E2F1* seems to be a promising therapy for impairing the proliferation of different tumor types and in other diseases where cell proliferation is a secondary effect, like vascular smooth muscle cell hyperplasia. Furthermore, the function of E2F1 in controlling the expression of other genes, such as *Cyclin A* and *Cyclin E*, and its overlapping function with other E2F members, namely E2F2 and E2F3, are controversial (Ohtani *et al.*,1995; DeGregori *et al.*, 1995; Takahashi *et al.*, 2000; Goto *et al.*, 2006; Kong *et al.*, 2007), thereby necessitating further characterization.

In this study, our aim was to develop short interfering RNAs for the impairment of cancer cell proliferation *in vitro*. *E2f1* was elected as the target, as its expression plays a key role in cell-cycle progression, besides being up-regulated in most types of tumor (Sherr, 1996; Dyson, 1998). We employed the rat glioma cell line, C6, as an *in vitro* cancer model, and showed that the shE2f1 (short hairpin RNA against E2f1 mRNA) is a potent tool for impeding cell proliferation, since it diminished C6 proliferation 3.5 to 4-fold. Furthermore, we also examined the effects of shE2f1 on the expression of two other members of the E2f family, *E2f2* and *E2f3*, to explore whether any cross-inhibition or compensatory mechanisms were occurring. The expression of *Cyclin A* and *Cyclin E* was also assessed to investigate E2f1 transcriptional regulation of these genes.

Three different shRNAs were designed for interference with the rat *E2f1* transcript at distinct regions (shE2f1A, B and C), and were inserted into the pBS/hU6-1 plasmid vector (generously provided by Dr. David Baltimore - *California Institute of Technology*, CA- USA – Qin *et al.*, 2003) yielding pBSE2f1A, B and C. As control, an additional vector (pBSshGFP) containing a shRNA against eGFP RNA (enhanced Green Fluorescent Protein) was also generated by using a previously validated target sequence described by Tiscornia *et al.* (2003) and Mousses *et al.* (2003). None of the target sequence shows any significant homology to other rat gene sequences. Therefore, synthetic oligonucleotides (Invitrogen) were designed (listed in Table 1) and cloned as described by Qin *et al.* (2003). The generated constructs were confirmed by sequencing, using 25 ng of the respective primers T3 and T7 (Stratagene) and the ABI Prism – Big Dye Terminator Cycle Sequencing Ready Reaction Kit, with an ABI377 sequencer, according to manufacturer's instructions (Perkin-Elmer).

The rat glioma cell line, C6 (ATCC CCL-107), was cultured in Dulbecco's Modified Eagle's minimum essential medium (DMEM high glucose), supplemented with 10% fetal bovine serum (FBS) and penicillin/streptomycin (Invitrogen) at 37 °C/5% CO_2_. C6 cells were plated at 80% confluence and co-transfected, using lipofectamine (Invitrogen) with 3 μg of a plasmid DNA mixture containing pBABEpuro and pBSshE2f1 -A, -B, -C or pBSshGFP (1:10, respectively), whereas pBS/hU6-1 derived plasmids were previously digested by *Xmn*I, according to manufacturer's protocol (BioLabs). After co-transfection, cells were selected using 400 ng/mL of puromycin. As the first step towards identifying the most effective pBSshE2f1, several clones, denominated C6shE2f1-A, -B, -C and C6shGFP, were obtained from each co-transfection and maintained in selective medium. Their genomic DNA was extracted with lysis buffer (100 mM Tris-HCl, pH 8.5; 5 mM EDTA; 0.2% SDS; 200 mM NaCl; 100 μg/mL of proteinase K), to verify the presence of pBS/hU6-1 derived plasmids by PCR using 25 ng of each of the primers T3 and T7 (Stratagene), according to manufacturer's instructions. pBS/hU6-1 was used as negative PCR control. Positive clones were selected and used in subsequent experiments.

In order to assess the proliferation-altering potential of each shRNA vector, the parental C6 cell line (triplicate) and different clones of C6shE2f1-A (3 clones), -B (5 clones), -C (3 clones) and C6shGFP (4 clones) were analyzed by a growth curve assay. At day zero, 5 x 10^4^ cells from each of the different clones (C6shE2f1-A; -B; -C; -shGFP), as well as the parental C6 cell line, were each seeded into 10 dishes, 35 mm in diameter, with DMEM supplemented with 5% FBS. At indicated times, each clone and the parental cells were sampled in duplicate. The final results shown in Figure 1 represent the average among clones of each type. The culture medium was replaced every two days. The data presented indicate that construct pBSshE2f1-B significantly impaired the proliferation of C6 cells when compared to controls. A comparative analysis at day 9 showed that C6shE2f1 B cell proliferation was 3.5 to 4 times lower than that observed in the controls, these cells remaining with only 30% of the proliferative capacity observed in C6shGFP cells.

Total RNA was extracted from C6 (parental cell line), and clones C6shGFP-6 (control cells), C6shE2f1-A22, C6shE2f-C3 and 2 different clones from C6shE2f1-B (B7 and B11), by using Trizol (Invitrogen) according to manufacturer's protocol (see Figure 1). This was carried out on the 7^th^ day of the growth curve, so as to ensure exponential growth and synchronized phases between the different cell-lines. This procedure was employed to minimize differences in *E2f1* expression due to the manner in which the cells were handled, and because E2f1 expression cycles during cell division. After RNA integrity was confirmed, each sample was treated with DNase I (Invitrogen) to avoid DNA contaminants, and purified by phenol/chloroform extraction before reverse transcription. An aliquot of 2 μg of RNA was used for first strand cDNA synthesis by priming with an oligo dT primer and using SuperScript II Reverse Transcriptase (Invitrogen) according to manufacturer's instructions. To control for DNA contamination of the cDNA samples, cDNA synthesis was performed in either the presence or the absence of reverse transcriptase. Samples were used as template for real time PCR amplification, where each cDNA was sampled in triplicate to detect *E2f1*, *E2f2*, *E2f3*, *Cyclin A and Cyclin E* gene expression. Real time PCR was performed in an ABI Prism 7700 Sequence Detection System (Applied Biosystems), according to manufacturer's guidelines. Expression of β*-actin* was assessed as an internal control, and used to calculate relative quantification as described by Pfaffl (2001). Each pair of primers was designed using Primer3 software, and their sequences are as follows: E2f1 F - 5' TGTGCCCTGAGGAAAGTG 3'; E2f1 R - 5' AAGGTTGGGGATGTGGAG 3'; E2f2 F - 5' AGTTCCTGTCCCCAATCCT 3'; E2f2 R - 5' GAGCCTGTCAATCTGTCTGTG 3'; E2f3 F - 5' GCCCATTGAGGTTTACTTGTG 3'; E2f3 R - 5' CCAGAGGAGAGAGGTTTGCT 3' (designed using as a template GenBank database E2f3 LOC291105 - E2f3 predicted from genome rat); Cyclin A F - 5' TTTGCCA TCGCTTATTGCT 3'; Cyclin A R - 5' TGTGGTGCTT TGAGGTAGGT 3'; Cyclin E F - 5' CTCGCTGCTTCT GCTTTGT 3'; Cyclin E R - 5' TGTGGGTCTGGATGTT GTG 3'; β-actin F 5'- ACCAACTgggACgATATggAgA AgA - 3'; and β-actin R 5'- TACgACCAgAggCATACA gggACAA - 3' (Invitrogen).

Detection of *E2f1* expression was performed in these samples by real time PCR to investigate shE2f1 efficiency. A comparative analysis of *E2f1* expression between one clone of each construct C6shE2f1 (-A22; -B7; -3C), C6 parental line and control clone C6shGFP-6 is presented in Figure 1b. The figure shows that E2f1 is more efficiently knocked down in C6shE2f1-B cells. These results are consistent with phenotypic observations.

Based on these results, two clones, C6shE2f1-B7 and -B11, were chosen to test *E2f1* expression by real time PCR. A comparative analysis of *E2f1* expression between two C6shE2f1-B clones (B7 and B11) and control clone C6shGFP6 is presented in Figure 2a. The data show that E2f1 was knocked down by as much as 77% in C6shE2f1-B clones. The data are in accordance with inactivation by RNA interference (RNAi) of other genes, as described in the literature (Shi, 2003). Therefore, knockdown E2f1 expression significantly impairs cell proliferation. These results are in disagreement with those of Humbert *et al.* (2000) and Wu *et al.* (2001), who suggest that E2f1 does not play a key role in cell proliferation, since cell division in the E2F1 knockout mouse is maintained. However, the cells used in their experiments were not malignant, as is C6, and so there was no exacerbated proliferation or the accumulation of genetic alteration.

We showed that knockdown of E2F1 by RNAi is a promising approach to impair unwanted cell proliferation, but we have not yet explored the impact of reduced E2F1 on apoptosis. In *E2f1* knockout mice, thymocytes revealed low levels of apoptosis and the animals had a high frequency of spontaneous tumor formation from different tissues (Field *et al.*, 1996; Yamasaki *et al.*, 1996). If designing a treatment strategy based on the induction of apoptosis, then shE2f1 may not be an appropriate option. Nevertheless, E2F1 is not the only factor involved in controlling the apoptosis pathway, since tumors can undergo apoptosis in the absence of its expression (Baudino *et al.*, 2003). Therefore, the choice of treatment could depend on the background of each tumor.

The E2F family includes 8 genes, most of which are involved in cell-cycle regulation. Only E2F1 to E2F3a are known to exert overlapping functions on inducing cell proliferation (Blais and Dynlacht, 2004). To test the influence that the lack of E2f1 may exert over the *E2f2*, *E2f3*, genes, their expression levels were also assessed (Figure 2a). Their expression was not affected in C6shE2f1-B clones, when compared to control cells. These results suggest that: i) shE2f1-B does not disrupt E2f2 and E2f3 expression, thereby proving its specificity; ii) E2f1 is not responsible for controlling E2f2 and E3f3 expression; iii) E2f2 and E2f3 do not compensate for the absence of E2f1 in cell proliferation, thus demonstrating that E2f1 was the major pro-mitotic effector under the present experimental conditions. Our data are in agreement with a recent study on HeLa cells, where the authors inhibited E2F1 by siRNA, and observed no effect on E2F2 expression (Goto *et al.*, 2006), and are also consistent with a study on double-knockout cells, where the authors found that E2F3 protein levels were unaffected by loss of E2F1/E2F2 (Li *et al.*, 2003). However, our findings are in contrast to those of Kong *et al.* (2007), as these authors conclude that the long-term loss of E2F activity leads to compensation by other family members. Nevertheless, our data were obtained from established clones and no compensatory effects were observed. A possible explanation for these different observations may be due to the different cell types that were utilized. Knockdown of E2F1 in cells with normal E2F1 expression may release a compensatory response in E2F2 and E2F3 expression, whereas in cancer cells, which usually over-express E2F1, knock down of this gene may not have a compensatory effect by other members of the family, thus being sufficient to impair proliferation.

Genes controlled by E2F1 have been described in a few studies where E2F1 was over-expressed. Cyclin A and Cyclin E were found to be over-expressed in response to E2F1, thereby demonstrating a direct correlationship between E2F1 and its targets (Ohtani *et al.*, 1995; Inoshita *et al.*, 1999, Takahashi *et al.*, 2000). The expression of these two genes was also assessed in a double-knockout model for E2f1/E2f2, where Cyclin A was down-regulated and Cyclin E was not significantly influenced (Li *et al.*, 2003). In contrast to these findings, Goto *et al.* (2006) revealed a different view by demonstrating that the lack of E2F1 does not negatively influence Cyclin A and Cyclin E. Because of the controversial function of E2F1 in controlling expression of genes involved in cell-cycle progression, we also analyzed *Cyclin A* and *Cyclin E* gene expression in C6shE2f1-B cells by real time PCR (Figure 2b). In accordance to E2F1 overexpression studies, *Cyclin A* was down-regulated in our cells when compared to controls, thus accompanying *E2f1* knockdown. However, this was not the case for *Cyclin E*, where expression was not significantly changed, when compared to control cells. This suggests that E2f1 does not control *Cyclin E* expression in C6 cells. However, continued expression of Cyclin E does not compromise the use of shE2F1-B in diminishing cell proliferation.

In conclusion, specific inactivation of E2f1 was sufficient to impair cell proliferation by 70%, and RNAi methodology seems to be an effective tool for targeting unwanted cell proliferation. Further investigation of these shRNAs in other cell-lines may provide additional information about this tool. Nevertheless, we have shown that shE2F1-B was capable of reducing the expression of E2f1, as well as impeding cell proliferation. With further development, shRNA against E2f1 may prove to be an interesting strategy in the treatment of proliferative diseases, such as cancer and other physiopathological conditions, including neointimal hyperplasia associated to cardiovascular derangements.

**Figure 1:**
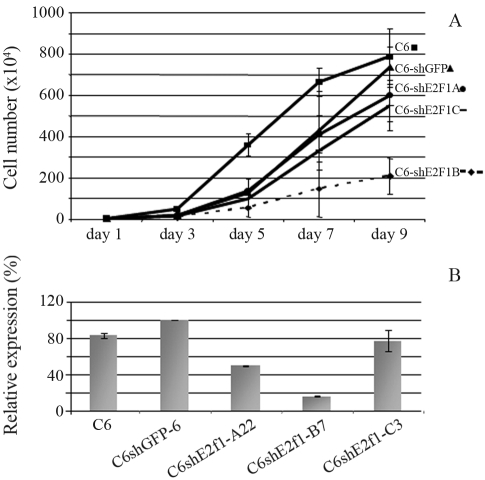
Phenotypic assay to assess activity of shE2f1. **(a)** In this growth curve, the data represent the mean cell number observed from parental C6 cells (triplicate), C6shGFP (4 clones), C6shE2f1-A (3 clones), C6shE2f1-B (5 clones) and C6shE2f1-C (3 clones) at the indicated time points. **(b)** Quantification of E2f1 transcript levels by real time PCR. E2f1 expression profile in cells C6, C6shGFP clone 6 (C6shGFP-6), and one clone from each of the different constructs C6shE2f1(-A22; -B7; -C3). Results were normalized by comparison to β-*actin* expression levels. cDNAs were synthesized from RNA obtained from synchronized cell cultures. The PCR assay is representative of the several assays performed.

**Figure 2:**
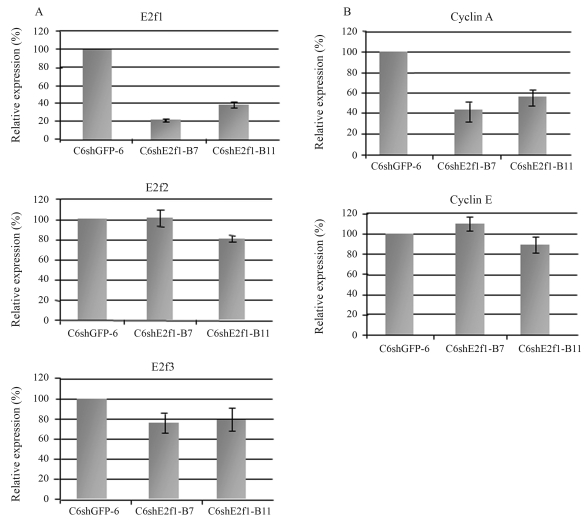
**(a)** E2f1, E2f2 and E2f3 expression profile in C6shGFP cells, clone 6, and C6shE2f1-B clones 7 and 11. **(b)** Expression profiles of E2f1 target genes, *Cyclin A* and *Cyclin E*, to evaluate their response to altered E2f1 levels. Expression of the indicated genes was determined as described legend of Figure 2. PCR reactions were performed three times.

## Figures and Tables

**Table 1:** Oligonucleotide sequences to construct pBS/hU6-1 encoding different shRNAs.

shRNAs name	Oligonucleotide sequence^a^
shE2f1-A sense (273)^b^	5'*ACC*gACCACCAAACgCTTCTTgTTCAAgAgACAAgAAgCgTTTggTggTCTTTTT*C* 3'
shE2f1-A antisense	5'*TCgAg*AAAAAgACCACCAAACgCTTCTTgTCTCTTgAACAAgAAgCgTTTggTggT 3'
shE2f1-B sense (426)	5'*ACC*gAATCATATCCAgTggCTATTCAAgAgATAgCCACTggATATgATTCTTTTT*C* 3'
shE2f1-B antisense	5'*TCgAg*AAAAAgAATCATATCCAgTggCTATCTCTTgAATAgCCACTggATATgATT 3'
shE2f1-C sense (246)	5'*ACC*gTCACgCTATgAgACCTCATTCAAgAgATgAggTCTCATAgCgTgACTTTTT*C* 3'
shE2F1-C antisense	5'*TCgAg*AAAAAgTCACgCTATgAgACCTCATCTCTTgAATgAggTCTCATAgCgTgA 3'
shGFP sense	5'*ACC*gCAAgCTgACCCTgAAgTTCTTCAAgAgAgAACTTCAgggTCAgCTTgCTTTTT*C* 3'
shGFP antisense	5'*TCgAg*AAAAAgCAAgCTgACCCTgAAgTTCTCTCTTgAAgAACTTCAgggTCAgCTTg 3'

a: underlined, 9-nucleotide spacer sequence. b: in parenthesis, starting nucleotide of shRNA target, based on GenBank accession number XM 230765.
